# Baroreflex Amplification and Carotid Body Modulation for the Treatment of Resistant Hypertension

**DOI:** 10.1007/s11906-020-1024-x

**Published:** 2020-03-12

**Authors:** Eline H. Groenland, Wilko Spiering

**Affiliations:** Department of Vascular Medicine, University Medical Center Utrecht, Utrecht University, 3508 GA Utrecht, The Netherlands

**Keywords:** Resistant hypertension, Baroreceptor, Carotid body, Device-based treatments, Baroreceptor amplification, Carotid body modulation

## Abstract

**Purpose of Review:**

Patients with true resistant hypertension (RH) are characterized by having high sympathetic activity and therefore potentially benefit from treatments such as baroreflex amplification (baroreflex activation therapy (BAT) or endovascular baroreflex amplification therapy (EVBA)) or carotid body (CB) modulation. This review aims at providing an up-to-date overview of the available evidence regarding these two therapies.

**Recent Findings:**

In recent years, increasing evidence has confirmed the potential of baroreflex amplification, either electrically (Barostim neo) or mechanically (MobiusHD), to improve blood pressure control on short- and long-term with only few side effects, in patients with RH. Two studies regarding unilateral CB resection did not show a significant change in blood pressure. Only limited studies regarding CB modulation showed promising results for transvenous CB ablation, but not for unilateral CB resection.

**Summary:**

Despite promising results from mostly uncontrolled studies, more evidence regarding the safety and efficacy from ongoing large randomized sham-controlled trials is needed before baroreflex amplification and CB modulation can be implemented in routine clinical practice.

## Introduction

According to the recent American and European guidelines for the management of arterial hypertension, resistant hypertension (RH) is defined as blood pressure (BP) that still exceeds the target despite the use of three antihypertensive medications in maximally tolerated daily doses with complementary mechanisms of action (a diuretic should be one component) [1–3] RH is an important cardiovascular risk factor and is estimated to be present in 5–20% of hypertensive patients [4, 5, 6•, 7]. Part of this population has so-called pseudo-resistant hypertension due to white-coat hypertension, improper BP measurement and/or medication non-adherence [7, 8]. After having excluded causes of pseudo-resistant hypertension, the true prevalence of resistant hypertension is likely to be < 10% of treated patients [2]. This true RH population might benefit from non-pharmacological device-based treatments.

Four major pathways contributing to the pathogenesis of resistant hypertension are sodium overload, arterial stiffness, endothelial dysfunction, and high sympathetic activity [9]. Most of the available device-based treatments have been designed to reduce the sympathetic nervous system outflow. Two device-based treatments target the sympathetic nervous system specifically at the level of the carotid sinus: baroreflex amplification therapy (electrically by barostimulator or mechanically by stent) and carotid body (CB) modulation.

In the past decades, these treatments have been increasingly studied in patients with RH. Comprehensive reviews about endovascular baroreflex amplification and CB modulation were already established in 2018 by van Kleef et al. [10] and Iturriaga [11], respectively. Of these reviews, the former emphasized both preclinical and clinical studies, the latter mainly focused on preclinical studies. Since 2018, there are ongoing research efforts to improve these innovative devices, with a keen focus on therapeutic efficacy and safety to best manage RH. Therefore, this review aims at providing an up-to-date overview with relevant clinical and experimental evidence of baroreflex amplification and CB modulation.

### Baroreceptors, Carotid Bodies, and Resistant Hypertension

**Fig. 1:**
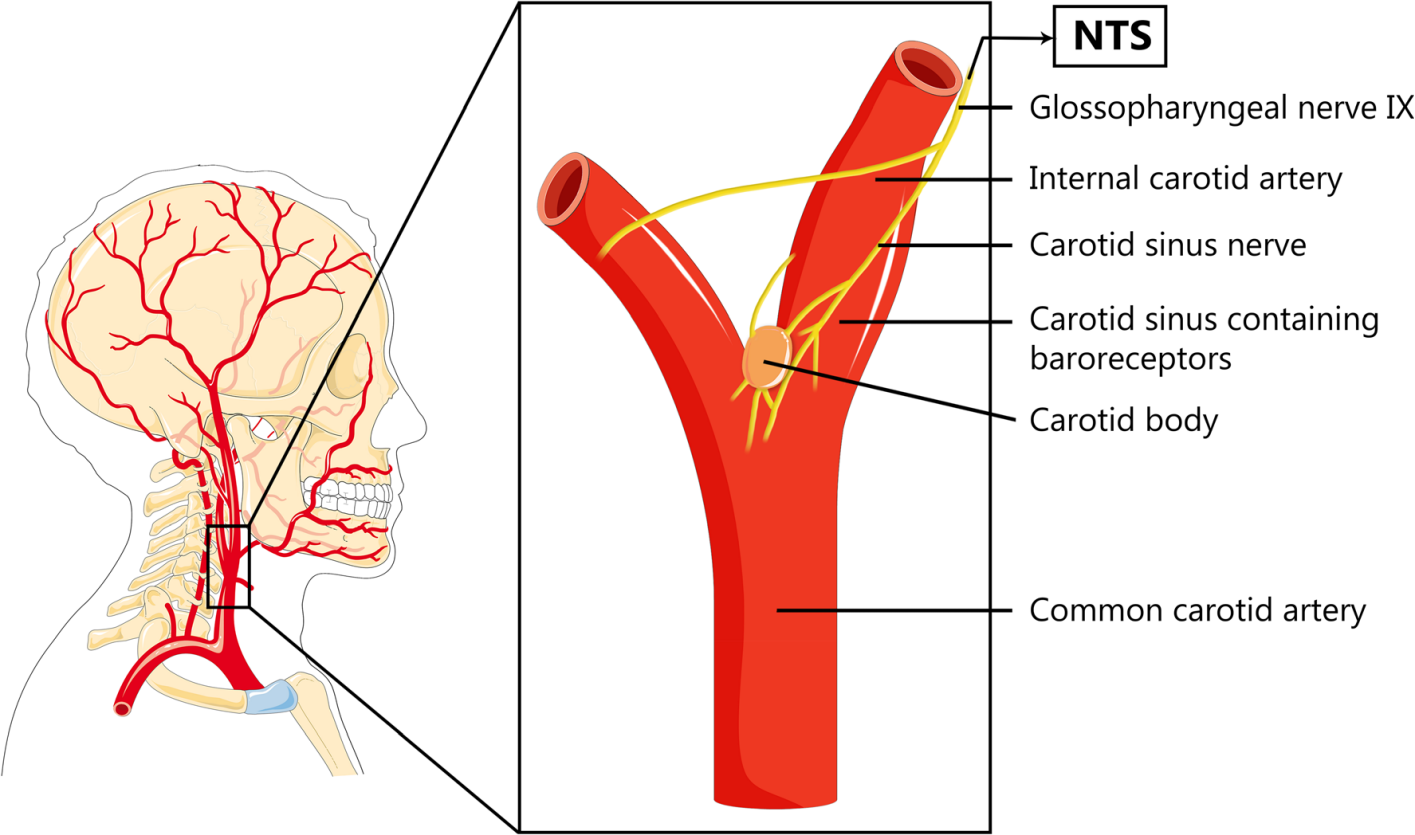
Carotid sinus and carotid body. Afferent nerve fibers travel from the baroreceptors located in the wall of the carotid sinus and chemoreceptors located in the carotid body via the glossopharyngeal nerve to the solitary nucleus of the medulla in the brainstem. This figure was created using Servier Medical Art templates, which are licensed under a Creative Commons Attribution 3.0 Unported License; https://smart.servier.com

Baroreceptors consist of stretch-sensitive fibers and are located in the area of the aortic arch and both carotid sinuses near the carotid bifurcation (see Fig. 1). Baroreceptors provide the afferent signals in a negative-feedback circuit in the medulla that maintains BP at normal levels. The receptors become active when the vessel wall stretches due to pulse waves by an increase in BP [12]. Subsequently, the signal is passed via the glossopharyngeal nerve to the nucleus tractus solitarius (NTS) in the dorsal medulla located in the brainstem. The primary afferent axons in the NTS synapse onto second-order neurons which in turn send excitatory projections to the GABAergic neurons in the region of the caudal ventrolateral medulla (CVLM). Then, the CVLM neurons synapse directly onto the excitatory rostral ventrolateral medulla (RVLM) neurons and inhibit the spontaneous activity of the RVLM [13]. As a consequence, the sympathetic tone is reduced and the parasympathetic tone is increased, finally leading to vasodilatation with consecutive normalization of the BP.

Baroreceptors are distinct from chemoreceptors, which are collections of highly specialized cells nested in the carotid and aortic bodies which are positioned near the baroreceptors at the carotid bifurcation. The chemoreceptors coordinate respiratory and arterial pressure changes during hypercapnia and chronic disturbances of acid–base balance. Activation of the carotid bodies by hypoxia drives, via the glossopharyngeal and vagus nerves, excitation in medullary presympathetic pathways (e.g., NTS). Via these pathways, the sympathetic nervous system is stimulated which will increase BP and ultimately aims at improving cerebral perfusion [14].

In hypertension, it is hypothesized that the baroreceptor sensitivity is reset to a higher operating pressure. Possible explanations for this phenomenon are direct damage to the receptors, a change in the coupling between the receptors and the vascular walls, genetically determined properties of the receptors, and decreased distensibility of the vascular walls in which the receptors are embedded [12].

### Baroreflex Amplification

#### Electrical Baroreflex Amplification

**Fig. 2:**
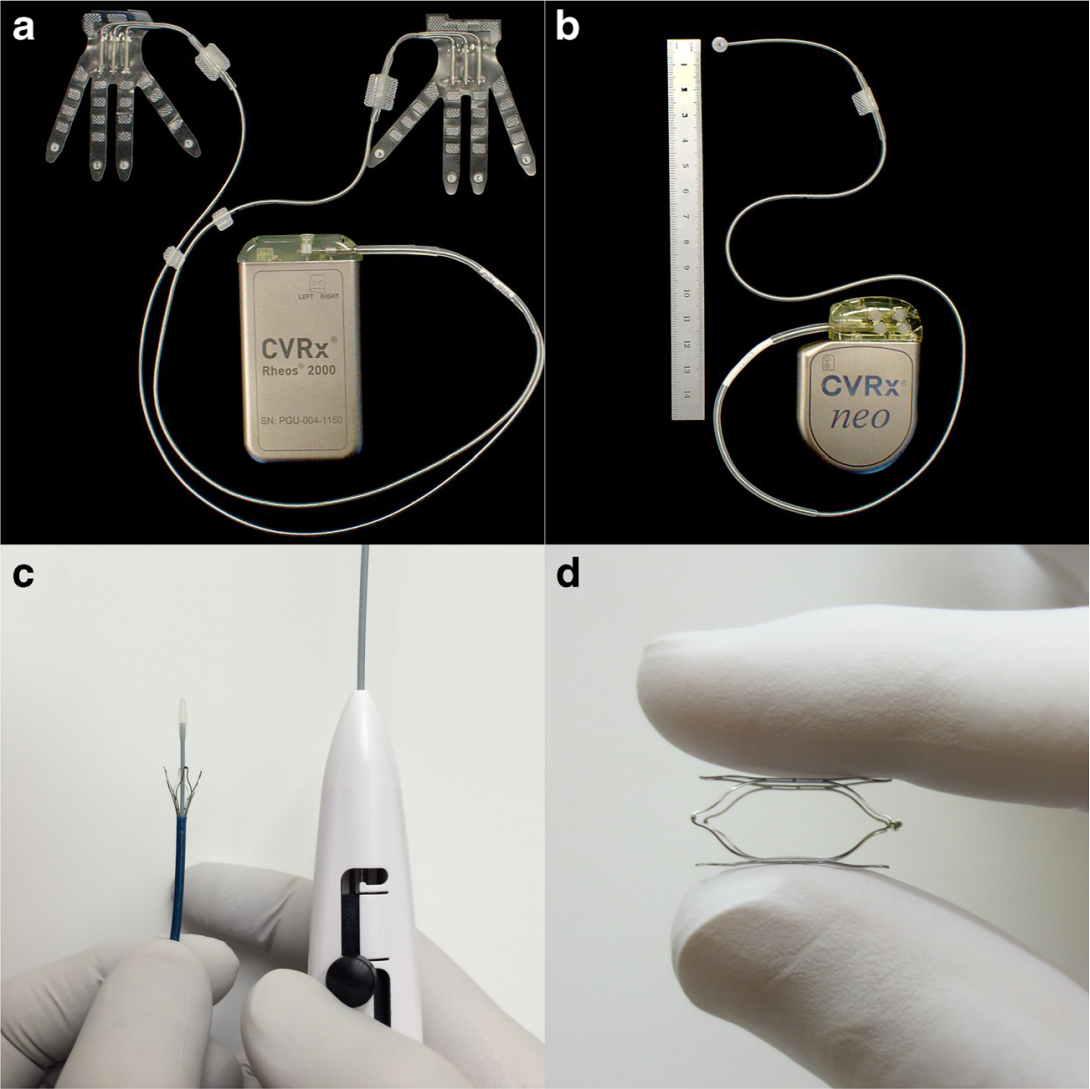
Devices for baroreflex amplification. **a**. The Rheos device (first generation) consists of bilateral electrodes and an implantable pulse generator. The bipolar electrodes in tripolar configuration are placed around both carotid sinuses and will electrically activate the baroreceptors. **b**. The Barostim neo device (second generation) consists of a unilateral electrode and lead and an implantable pulse generator. The electrode is sutured onto the arterial wall and will stimulate the carotid sinus. **c**. The MobiusHD is delivered by a catheter which is introduced over a guidewire via the femoral artery. **d**. The self-expanding nitinol MobiusHD device which is implanted in the internal carotid artery for amplification of the carotid baroreceptor signal

Early studies in 1960 and 1970 showed favorable results regarding BP reduction [15–17]. However, baroreflex stimulation trough electrodes wrapped around the carotid sinus nerve was halted due to technical difficulties with electrode implantation, adverse effects related to nerve injury and the introduction of more effective and better tolerated antihypertensive drugs [18]. This remained unchanged until 2001 when CVRx, Inc. (USA), a start-up company, was founded and introduced an improved carotid baroreceptor pacemaker addressing previous limitations: the first-generation electrical carotid sinus stimulator (Rheos)(see Fig. 2a) [19].

The Rheos device consisted of two pulse generators that were surgically implanted around the carotid bulbs bilaterally along with a pulse generator placed in a subcutaneous pocket in the chest [19]. Animal studies (in dogs) showed that 7 days continuous baroflex activation by the Rheos device resulted in a substantial and sustained reduction in mean arterial pressure, heart rate, and norepinephrine levels, without a compensatory increase in plasma renin activity [20].

The open-label, non-randomized US Rheos Feasibility phase II trial studied the response of ten patients with RH to baroreflex activation therapy using the Rheos system [21]. This study showed a mean reduction in office systolic BP of 41 mmHg (range, 22–104 mmHg; *p* < .001) with a peak response at 4.8 V (*p* < .001) without significant bradycardia or bothersome symptoms [21].

Subsequently, the multicenter non-randomized feasibility Device-Based Therapy in Hypertension (DEBuT-HT) study assessed BP reduction and safety at 3 months post-implantation in 45 patients with a BP of ≥ 160/90 mmHg while on treatment with at least two antihypertensive drugs [22]. The study demonstrated a decrease in mean office BP of 21 ± 4/12 ± 2 mmHg after 3 months and 33 ± 8/22 ± 6 mmHg after 2 years [22]. Despite these encouraging results, data regarding the durability of the antihypertensive action obtained in from a randomized trial was lacking.

To overcome this limitation, the double-blind randomized Rheos Pivotal trial was designed [23]. The Rheos Pivotal trial assessed the safety and efficacy of carotid baroreflex activation therapy in 265 patients with RH [23]. One month after implantation of the Rheos device each patient was randomly assigned to either immediate initiation of baroreceptor stimulation (group A, *n* = 181) or delayed initiation until the 6-month follow-up (group B, *n* = 84). In this trial, five co-primary endpoints were pre-specified; acute and sustained efficacy as well as procedural, BAT, and device safety. The acute efficacy endpoint (proportion of subjects that achieve at least a 10 mmHg drop in systolic BP at month 6 compared with baseline, with a superiority margin of 20%) was reached in 54% of the subjects in group A and 46% of the subjects in group B, which was not statistically significant. Moreover, the criteria for procedural safety were not met as 25.5% of the patients suffered from surgical complications wound complications or nerve damage [23]. However, the mean reduction in systolic BP after 6 months compared to baseline was 16 ± 29 mmHg in the stimulated group versus 9 ± 29 mmHg in the control group (*p* = 0.08) and long-term follow-up of 22 to 53 months of the Rheos Pivotal trial alone showed sustained reduction of mean systolic BP of 35 ± 31 mmHg versus pre-implantation among responders [24].

A combined long-term follow-up study of the US Rheos Feasibility Trial, the DEBuT-HT Trial, and the Rheos Pivotal Trial showed sustained effect on BP after 6 years of follow-up (mean office BP 179 ± 24/103 ± 16 mmHg before treatment and 144 ± 28/85 ± 18 mmHg after treatment) [25•]. Major drawbacks for use of the first-generation Rheos device in standard clinical practice were its invasiveness and short battery life which needs replacement every 3 to 5 years.

On the basis of these mixed results, a considerable amount of surgical complications and nerve injury the US Food and Drug Administration (FDA) did not approve the Rheos system for the treatment of RH. Therefore, the Rheos system is not available anymore.

The second-generation device (Barostim neo) (see Fig. 2b), which is approved and clinically applied in Europe, uses a smaller, one-sided unipolar disk electrode to decrease invasiveness and to improve battery life. The uncontrolled, open-label Barostim neo trial was the first study investigating the efficacy of the device [26]. This trial included 30 patients with RH from seven centers in Europe and Canada and showed a mean reduction in office systolic and diastolic BP of 26.0 ± 4.4 and 12.4 ± 2.5 mmHg, respectively, at 6 months. Interestingly, a subset of six individuals who already underwent renal denervation demonstrated similar reductions in BP after implantation of the Barostim neo implying that baroreflex activation works through mechanisms broader than inhibition of renal sympathetic nerve activity. Moreover, within the first 30 days after implantation of the Barostim neo 90% of patients were free from system- or procedure-related events, compared with 75% in the Rheos Pivotal Trial and the few events that did occur resolved without sequelae. A few years later, the device was further studied in a single-arm study among 51 patients with RH. This study reported a significant decrease in mean 24 h ambulatory systolic BP (from 148 ± 17 to 140 ± 23 mmHg; *p* < 0.01) and diastolic BP (from 82 ± 13 to 77 ± 15 mmHg; p < 0.01) at 6 months after the procedure [27].

Another study that investigated the sympathetic vasoconstrictor tone and BP response of the Barostim neo device in 18 patients with RH reported that stimulation with intensities that produced tolerable adverse effects in the short term resulted in a mean decrease in office systolic BP of 16.9 ± 15.0 mmHg (*p* = 0.002) [28]. However, 12 patients (66.7%) experienced stimulation-related side effects such as jaw or neck pain, globus, or swallowing sensation, coughing, or voice problems. In these patients, stimulation intensity for the long-term treatment, therefore, had to be reduced. This reduced stimulation intensity resulted in a significant reduction in efficacy with a mean decrease in office systolic BP of only 6.3 ± 7.0 mmHg (*p* = 0.028) [28].

Long-term follow-up of patients treated in the Barostim neo trial showed a sustained BP-lowering effect (mean office systolic BP reduction of 26.2 ± 35.2 mmHg) of unilateral baroreflex amplification therapy and an acute effect of device deactivation and reactivation on BP after 16.5 months of baroreflex amplification therapy, supporting the efficacy of baroreflex amplification therapy [29]. The largest reported cohort treated with the Barostim neo so far demonstrated long-term BP reduction in 60 patients with RH [30]. Patients were defined as responders if they showed a reduction in systolic BP of ≥ 10 mmHg in office and/or ≥ 5 mmHg in ambulatory BP monitoring (ABPM). Twenty-four months after implantation of the Barostim neo, 35 patients (70%) could be classified as a responder according to office measurements and 21 patients (46%) could be classified as a responder according to the ABPM criterion. Overall, 50% of the patients reached target office SBP of 140 mmHg or below [30].

Baroreflex activation therapy also seems to be effective for lowering blood pressure in RH patients with renal failure. Wallbach et al. studied 23 chronic kidney disease patients with RH treated with the Barostim neo. They found a mean office BP fall of 17/9 mmHg as compared to 1/1 mmHg fall in 21 patients in the control group (standard medical management) after 6 months (*p* < 0.01) [31]. Beige et al. investigated the effect of baroreflex activation therapy in seven patients with end-stage renal disease and RH [32]. They found a significant decrease in office systolic BP from 194 ± 28 to 137 ± 16 mmHg (*p* < 0.01).

Finally, the Barostim neo has also shown to be safe and effective in patients with heart failure and reduced ejection fraction (HFrEF) [33–35]. The Baroreflex Activation Therapy for Heart Failure (BeAT-HF) study is a phase III multi-center, non-blinded randomized controlled trial in HFrEF patients with NYHA class III and an ejection fraction of ≤ 35% [36]. Patients were randomized to receive either the Barostim neo plus optimal medical management or optimal medical management alone. The expedited phase of this trial included 408 patients and focused on patient-centered symptomatic outcomes such as quality of life assessed by the Minnesota Living with Heart Failure Questionnaire (MLWHF) (decrease of 5 points is considered clinically meaningful) and exercise capacity assessed by the 6-min hall walk distance (6MHWD) score (increase of at least 25 m is considered clinically meaningful) [37•]. The study showed that the Barostim neo was safe and significantly improved quality of life score (14 points more than the control group (Δ = − 14 [95% CI -19, -9]), exercise capacity (60 m greater increase in six-minute hall walk distance in BAT versus control (Δ = 60 [95% CI 40, 80]), and NT-proBNP (25% greater reduction in NT-proBNP compared to the control group (inverse transformed Δ = − 25% [95% CI -38%, -9%]) [37•]. These significant differences in treatment effect were observed despite an increase in the number of medications in the control arm. However, BeAT-HF was not a blinded study, therefore it has to be acknowledged that the previously described effects on patient-centered symptomatic endpoints may be subject to placebo effects. The manuscript draft of this trial is currently under review for publication in The Lancet [37•].

#### Ongoing Research

Studies to date have shown promising results with the Barostim neo device. However, randomized sham-controlled trials are needed to confirm the effects of the Barostim neo on both office and 24-h ABPM. A prospective cohort study is currently being conducted in Germany. Up to 500 subjects will be enrolled at up to 50 sites and the follow-up duration is 3 years. Furthermore, two randomized studies are currently in progress; the Nordic BAT study, a randomized double-blind trial which aims to enroll 100 patients (clinicaltrials.gov: NCT02572024) and the Economic Evaluation of Baroreceptor STIMulation for the Treatment of Resistant HyperTensioN (ESTIM-rHTN) study, a randomized open-label trial which aims to enroll 128 patients (clinicaltrials.gov: NCT02364310). The latter will compare the cost-effectiveness of carotid barostimulation using the Barostim neo system with usual care in patients with RH and is expected to be completed in early 2021. After personal contact with the principal investigators of both trials, we confirmed an inclusion status of 7/100 and 84/128, respectively. The multicenter randomized Barostim neo Hypertension Pivotal Trial which started in 2013 is currently suspended because company resources will only allow adequate oversight for one pivotal trial at a time (clinicaltrials.gov: NCT01679132).

### Mechanical Baroreflex Amplification

An alternative approach to baroreceptor amplification therapy is to use an endovascular implant to increase circumferential and longitudinal wall strain at the level of the carotid baroreceptors, potentially resulting in activation of the baroreflex and lowering of BP. The MobiusHD (Vascular Dynamics, Mountain View, CA, USA) is a nitinol self-expanding rectangular cuboid implant (see Fig. 2c and d) which was first tested in dogs showing an acute reduction in BP of 50/30 mmHg [38••]. This effect sustained for 6 h without resetting or change in other hemodynamic effects.

The CALM-FIM (Controlling and Lowering BP With The MobiusHD – First In Man) study was the first one to study the performance of the Mobius HD in humans [38••]. This study is a prospective multicenter single-arm safety study among 30 European and US adult patients with RH. The primary endpoint was the incidence of serious adverse events at 6 months. Secondary endpoints included changes in office and 24-h ambulatory BP. During 6 months of follow-up, four patients (13%) developed serious adverse events, including hypotension (*n* = 2), worsening hypertension (*n* = 2), leg claudication (*n* = 1), and wound infection (*n* = 1). Reductions in mean office and 24-h ambulatory BP at 6 months were 24/12 mmHg (13–34/6–18) and 21/12 mmHg (14–29/7–16), respectively (*p* < 0.001) [38••]. The long-term results of this study are expected to be published soon.

Currently, the open-label single-arm CALM-DIEM study investigating the safety and efficacy of the MobiusHD in Europe, is enrolling 200 patients. A substudy of CALM-DIEM was designed to determine the mechanism of action of the MobiusHD device by studying the effect of MobiusHD implantation on sympathetic activity and baroreflex sensitivity using microneurography and functional magnetic resonance imaging (fMRI).

#### Ongoing Research

Despite these promising results randomized, double-blind, sham-controlled clinical trials are needed to confirm the safety and efficacy of the MobiusHD device. Therefore, the CALM-2 study, a multicenter, prospective, randomized, double-blind, sham-controlled pivotal study in Europe and the USA is currently enrolling 300 patients with RH (clinicaltrials.gov: NCT03179800). Another sham-controlled trial, the CALM-START study, aims to eliminate the confounding effects of antihypertensive medications on the efficacy of the MobiusHD device and therefore evaluates the effects of the MobiusHD after washout of such medications (clinicaltrials.gov: NCT02804087).

### Carotid Body Modulation

CB hyperactivity increases central sympathetic drive and thus contributes to hypertension through direct increases in renal neurogenic sodium retention and increases in renin secretion, as well as neurogenically mediated increases in arterial resistance [39]. Therefore, CB resection is proposed as a target for the treatment of RH [40].

CB resection was historically developed as a possible treatment for dyspnea in patients with asthma and chronic obstructive pulmonary disease (COPD). Besides the beneficial effects of CB resection on asthmatic symptoms Nakayama et al. also reported sustained BP reductions after CB resection [40, 41]. They reported BP findings in 29 patients in a single series from 1940s to 1960s: a reduction in systolic BP from 170 mmHg preoperative to 130 mmHg at 5 days postoperative and this reduction was maintained until the end of the study (6 months) [40, 41]. Additionally, Winter and Whipp studied the effects of bilateral CB removal in a cohort of 32 patients with chronic obstructive pulmonary disease and asthma [40, 42]. Although not the primary purpose of surgery in these patients, acute reduction in both systolic and diastolic BPs of about 20 mmHg was observed [42].

These promising findings led to the design of a proof-of-principle study evaluating the safety and feasibility of unilateral CB resection in 15 patients with RH [43••]. This study showed acceptable safety and feasibility, but failed to show a statistically significant reduction in office or ambulatory systolic BP at 1, 3, 6, and 12 months of follow-up compared to baseline. Moreover, a control group was lacking and the number of patients was limited to 15. In addition, a proof-of-concept, safety, and feasibility trial regarding the effect of CB resection on sympathetic tone in ten male patients with moderate heart failure was also not able to show significant changes in mean office BP either 1 or 2 months post-CB resection (82.5 ± 3.7 vs. 85.3 ± 3.2 mmHg, *p* = 0.19 and 85.2 ± 4.1 vs. 84.6 ± 3.0 mmHg, *p* = 0.91, respectively) (secondary outcome) [44•].

Due to its invasiveness, surgical resection of the CB is unlikely to attract widespread acceptance by patients and treating physicians. In contrast, a catheter-based interventional approach, which is feasible either directly via arterial access through the carotid artery or via a transjugular access, appears justifiable if an acceptable safety profile and a clear signal for BP-lowering efficacy can be found.

In 2018, an abstract including the preliminary results of a proof-of-principle cohort study using endovascular venous catheters for right-sided CB ablation in patients with RH was published [45•]. Data from 27 patients showed that unilateral CB ablation resulted in a mean 24-h ambulatory BP reduction of 9.1 ± 13.5/6.7 ± 8.7 mmHg at 6 months with similar reductions observed already at 1 and 3 months follow up. One hospitalization for hypertensive crisis and one transient ischemic attack were reported as serious adverse events. No final results have been published yet.

#### Ongoing Research

Prospective randomized clinical trials are needed to investigate the performance of CB ablation in the treatment of RH. However, currently, no such studies are registered on Clinicaltrials.gov.

### Device-Based Treatments for Non-adherent Patients?

Non-adherence is a major issue among patients with apparent RH and is associated with increased risk of cardiovascular disease, hospitalization, and healthcare costs [46].

Some experts suggest that patients who do not wish to take drugs lifelong or who do not adhere to antihypertensive drugs due to adverse effects might also be potential candidates for device-based treatment [47]. However, based on the mean BP reductions outlined in this review, device-based treatment by baroreflex amplification or carotid modulation will be unlikely to achieve complete BP control without concomitant use of antihypertensive drugs. Therefore, taking antihypertensive medication will remain part of the treatment regimen. Moreover, one could question whether these patients, being non-adherent for antihypertensive drugs, will adhere to other treatment requirements accompanying these expensive irreversible device-based treatments (e.g., antiplatelet therapy after MobiusHD implantation or follow-up visits)?

Whereas device-based treatments will probably reduce BP they are unlikely to affect erroneous adherence behavior. Therefore, in non-adherent patients, educational measures, behavioral interventions and e-health interventions would probably be a better strategy to start with.

## Conclusions

In recent years, more attention is being paid to identification of true RH and thereby identification of patients who might benefit most from device-based treatments. Since sympathetic overactivity plays a crucial role in these patients, device-based treatments targeting the sympathetic nervous system such as baroreflex amplification and CB modulation are actively being studied.

This review summarized the available evidence regarding carotid-based treatments and showed that baroreflex amplification either via the Barostim neo system or by endovascular MobiusHD stent placement and CB modulation via endovascular venous catheters holds promise as novel therapies to supplement, but not substitute, pharmacological treatment for patients with true RH.

However, before implementation in clinical practice, the current evidence has to be confirmed by results from ongoing randomized, sham-controlled trials. Furthermore, future research should also address to what extent the BP reduction by these treatments reduce cardiovascular events and mortality.
